# Biochemical Fingerprint of Early Healing After Enamel Matrix Derivative Application Using a Flapless Approach: A Randomized Clinical Trial

**DOI:** 10.3390/ijms26188766

**Published:** 2025-09-09

**Authors:** Federica Romano, Giacomo Baima, Morta Stasikelyte, Ahmad Bebars, Anna Brusamolin, Francesco Franco, Giovanni Nicolao Berta, Mario Aimetti

**Affiliations:** 1 Department of Surgical Sciences, CIR Dental School, University of Turin, 10126 Turin, Italy; stasikelyte@gmail.com (M.S.); ahmad_bebars@hotmail.com (A.B.); anna.brusamolin@edu.unito.it (A.B.); 2 Department of Clinical and Biological Sciences, University of Turin, 10043 Orbassano, Italy; francesco.franco@unito.it (F.F.); giovanni.berta@unito.it (G.N.B.); mario.aimetti@unito.it (M.A.)

**Keywords:** biomarkers, gingival crevicular fluid, intrabony defects, minimally invasive non-surgical procedures, periodontal regeneration

## Abstract

This study aimed to investigate the effect of enamel matrix derivatives (EMD) on the early healing biomarkers’ expression following flapless treatment. Thirty-eight patients with residual deep intrabony defects after steps 1 and 2 of periodontal therapy were randomly assigned to the test (flapless with EMD) or control group (flapless alone). Periodontal parameters were recorded at baseline and 6 months after treatment. Gingival crevicular fluid (GCF) was collected at baseline and 2 weeks after treatment to quantify the levels of biomarkers related to epithelial healing (epidermal growth factor, EGF), connective tissue healing (matrix metalloproteinase-8 [MMP-8], fibroblast growth factor [FGF], transforming growth factor-β [TGF-β]), and bone formation (osteoprotegerin [OPG]). The test group showed a significant reduction in MMP-8 levels (*p* = 0.039), along with significant increases in EGF (*p* < 0.01), FGF (*p* < 0.01), and OPG (*p* < 0.01). The control group demonstrated a significant decrease in MMP-8 (*p* = 0.010). No significant changes in TGF-β levels were observed in either group. At 6 months, the test group exhibited significantly greater reductions in probing pocket depth and clinical attachment level compared to the control group. This study is the first to characterize the biochemical changes following flapless treatment with EMD. These preliminary findings suggest that EMD may enhance early wound healing by modulating the expression of key regenerative biomarkers.

## 1. Introduction

According to European Federation of Periodontology guidelines, periodontal regeneration is recommended for residual intrabony defects ≥ 3 mm [1,2], with wound stability being a critical prerequisite for success [3,4]. Minimally invasive surgical techniques (MIST) have been developed to support this by minimizing trauma, preserving soft tissue integrity, and ensuring primary closure—leading to improved clinical and radiographic outcomes [4,5,6]. The adoption of magnification systems and microsurgical instruments has further extended the principles of MIST to non-surgical periodontal therapy [7,8]. Recently, the flapless approach has emerged as an evolution of the minimally invasive paradigm, avoiding incisions altogether and maintaining interdental soft tissue, thereby enhancing clot stability and early healing [9,10]. When combined with enamel matrix derivative (EMD), flapless therapy shows additional clinical benefits [10,11,12]. EMD, derived from porcine enamel proteins, promotes regeneration by stimulating periodontal ligament and osteoblast precursor cell proliferation, mesenchymal cell differentiation into cementoblasts and angiogenesis [13,14]. It also regulates growth factor expression in periodontal ligament cells and gingival fibroblasts, while exerting anti-inflammatory effects [15,16].

Previous studies have identified several growth factors, including transforming growth factor-β (TGF-β), epidermal growth factor (EGF), fibroblast growth factor (FGF) and osteoprotegerin (OPG), as potential contributors to periodontal regeneration [17,18], EGF primarily supports re-epithelialization, while FGF, TGF-β and OPG are involved in periodontal ligament and alveolar bone regeneration by stimulating angiogenesis, fibroblast proliferation, collagen production, and inhibiting osteoclast activity [19,20,21,22]. Conversely, elevated or sustained expression of pro-inflammatory mediators, such as matrix metalloproteinase (MMP)-8, has been associated with connective tissue degradation, highlighting the importance of a well-regulated molecular environment for effective periodontal healing [23,24,25]. However, the precise roles and timing of these factors during the early phases of wound healing remain poorly understood. Although EMD has demonstrated regenerative potential, its specific influence on multiple key biomarkers in the immediate post-treatment phase following flapless treatment has not been thoroughly investigated. Therefore, this trial aimed to evaluate the adjunctive effect of EMD on the expression profile of early regenerative markers in the gingival crevicular fluid (GCF) of deep intrabony defects treated with a flapless approach. As a site-specific biological fluid, GCF reflects the local inflammatory status of periodontal tissues, making it a valuable medium for monitoring host response during periodontal therapy [26,27,28,29].

Based on the established biological properties of EMD, we hypothesized that its local application in conjunction with a flapless procedure would reduce the inflammatory response and enhance the expression of key regenerative biomarkers during the early healing phase.

## 2. Results

### 2.1. Patients’ Characteristics

A total of 47 patients were consecutively screened for eligibility. Of these, 6 did not meet the inclusion criteria and 3 declined to participate in the study. Ultimately, 38 patients, each contributing one intraosseous periodontal defect, were enrolled and randomly allocated to the test (flapless + EMD) or control group (flapless alone). The flapless procedure was performed in 19 subjects (12 males; mean age: 55.6 ± 7.6 years) and the flapless + EMD procedure was applied in the remaining 19 subjects (8 males, mean age: 53.1 ± 11.6 years). No participants discontinued the study, and complete data were available for statistical analysis.

Patient and defect characteristics are presented in Table 1. In the test group, the distributions of intrabony defects by tooth type were 47.4% anterior, 15.8% premolar and 36.8% molar. In the control group, the corresponding distribution was 36.8% anterior, 21.1% premolar, and 42.1% molar. The two experimental groups were well balanced with respect to demographic and defect-related characteristics.

### 2.2. Clinical Outcomes

No adverse events were observed at any of the treated sites. The clinical periodontal parameters for both study groups at baseline and at the 6-month follow-up are summarized in Table 2. The site-level presence of bacterial plaque (PI) showed a slight but non-significant reduction over time in both groups (from 26.3% to 10.5% in the test group and from 31.6% to 15.8% in the control group; *p* > 0.05). In contrast, the proportion of sites with bleeding on probing (BoP) significantly decreased in both groups at 6 months post-treatment (from 89.5% to 21.1% in the test group and from 94.7% to 26.3% in the control group; both *p* < 0.001).

Both treatment approaches resulted in a statistically significant reduction (*p* < 0.001) in mean probing pocket depth (PPD) and clinical attachment level (CAL), accompanied by a slight, clinically negligible increase in gingival recession (REC). At 6 months, mean PPD and CAL in the test group significantly decreased to 4.0 ± 1.2 and 4.8 ± 1.5 mm, respectively. In the control group, corresponding values were 4.8 ± 1.3 and 5.8 ± 1.4 mm. At 6 months, both PPD and CAL values were significantly lower in the test group compared to the control group (*p* < 0.05), while the increase in REC showed no statistically significant differences between the groups.

### 2.3. Biomolecular Outcomes

**Figure 1:**
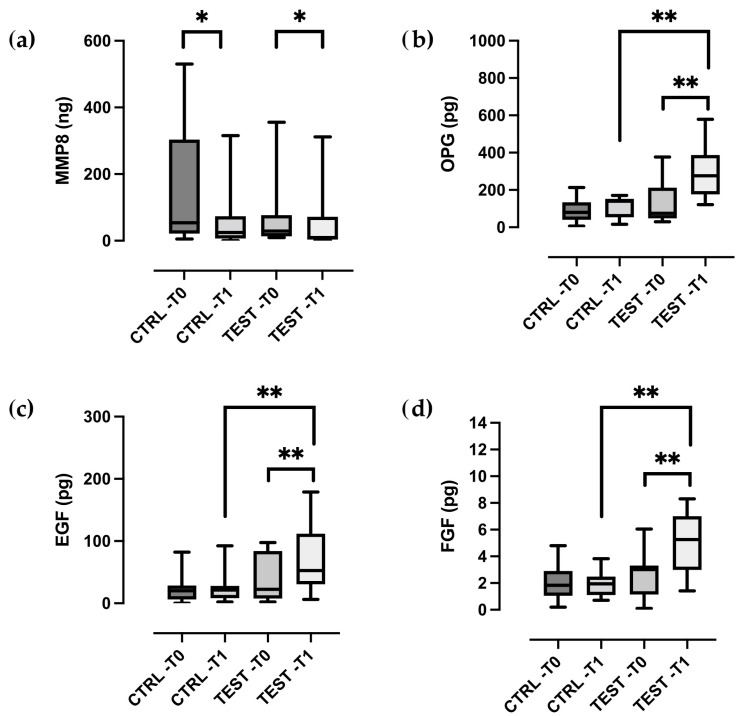
Box- and-whisker plots showing the total amount of (**a**) MMP-8, (**b**) OPG, (**c**) EGF, (**d**) FGF in gingival crevicular fluid of the control and test groups at baseline (T0) and two weeks post-treatment (T1). The box represents median, 25% and 75% percentiles, the whiskers represent data within 10% and 90% percentiles. * *p* < 0.05, ** *p* < 0.01.

At baseline, no significant differences were observed between the test and control groups for any of the inflammatory mediators analyzed (*p* > 0.05). At two weeks after treatment, test sites demonstrated a statistically significant reduction in the total amount of MMP-8 (*p* = 0.039), along with significant increase in EGF, FGF, and OPG levels in GCF (all *p* < 0.01) (Figure 1a–d). In contrast, control sites showed a significant decrease in MMP-8 (*p* = 0.011), but no significant changes in the levels of the other biomarkers (Figure 1a–d).

**Figure 2:**
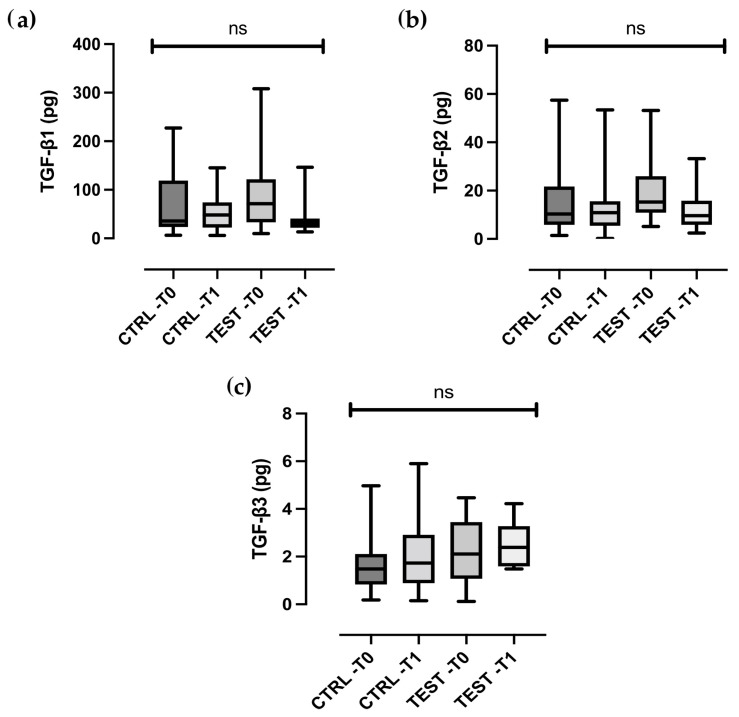
Box- and-whisker plots showing the total amount of (**a**) TGF-β1, (**b**) TGF-β2, (**c**) TGF-β3 in gingival crevicular fluid of the control and test groups at baseline (T0) and two weeks post-treatment (T1). The box represents median, 25% and 75% percentiles, the whiskers represent data within 10% and 90% percentiles. ns, not statistically significant.

TGF-β levels remained largely unchanged in both groups (Figure 2a–c). Statistically significant difference between the test and control groups were observed for all biomolecular markers, except MMP-8 and TGF-β (all *p* < 0.01).

As part of the explorative analysis, correlations between clinical and biomolecular parameters were also assessed. A significant negative correlation was found between MMP-8 levels in the GCF at 2 weeks and both PPD reduction (r = 0.568, *p* < 0.001) and CAL gain (r = 0.469, *p* = 0.004) at 6 months. Conversely, OPG levels at 2 weeks showed a positive correlation with both PPD reduction (r = 0.499, *p* = 0.012) and CAL gain (r = 0.511, *p* = 0.018). Additionally, EGF (r = 0.361, *p* = 0.036) and FGF (r = 0.500, *p* = 0.021) levels were positively correlated with CAL gain at 6 months.

## 3. Discussion

This randomized clinical trial aimed to evaluate the early molecular response induced by EMD application during flapless periodontal treatment. Using a multiplex immunoassay, we quantified selected growth factors and matrix degradation markers in GCF collected at two weeks post-treatment. This technique facilitates the simultaneous analysis of multiple biomarkers in minimal volumes of liquid. Owing to the limited sample volume, conventional biochemical methods are often insufficient for quantifying adequate levels of multiple analytes in GCF obtained from individual sites.

We found significant differences for three biomarkers (FGF, OPG and EGF) between samples from patients with and without EMD application following the flapless approach, while both groups experienced a reduction in MMP-8 levels. These findings suggest that EMD application influences not only the resolution of local inflammatory burden, as reflected by MMP-8 reduction, but also stimulates the expression of mediators associated with epithelial repair, connective tissue remodeling, and bone metabolism compared to the flapless procedure alone.

Periodontal therapy is known to affect significantly the biomolecules expression pattern in gingival tissues and the GCF, also after the non-surgical initial phase [23,28,29,30,31]. However, the specific modulatory effects of biologic inductors, such as EMD, on this molecular response remain underreported and insufficiently characterized. The increase in EGF and FGF levels following EMD application aligns with in vitro studies reporting the stimulatory effect of EMD on fibroblasts and periodontal ligament cells [14,32,33]. The present data provide further evidence of this effect in the early post-treatment phase. Since both EGF and FGF are involved in epithelial proliferation and extracellular matrix synthesis, their upregulation may reflect the activation of early tissue repair processes [27,34]. Nevertheless, it is not possible to establish whether these molecular changes directly contribute to clinical tissue regeneration or reflect secondary wound responses [23].

The increase in OPG levels observed in the EMD-treated sites is of particular interest, given the OPG role in inhibiting osteoclastogenesis through RANKL antagonism. While previous research has described EMD increasing OPG synthesis in vitro [14], its early modulation following EMD application had not been previously reported in humans. Whether this finding indicates an effect of EMD on early bone turnover or reflects modulation of the inflammatory environment remains to be clarified.

Both treatment groups exhibited significant reductions in MMP-8 levels at two weeks. As MMP-8 is a collagenolytic enzyme associated with periodontal tissue degradation, its decrease likely reflects the resolution of active inflammation following mechanical debridement and wound closure [35,36,37]. The absence of additional MMP-8 suppression in the EMD group suggests that the primary effect of EMD during early healing may not involve further modulation of inflammatory protease activity [38]. In this case, only total MMP-8 was assessed, and we cannot therefore infer on the role of EMD on its active form.

TGF-β levels remained stable in both groups. This result contrasts with in vitro studies suggesting that EMD induces TGF-β expression in fibroblasts [39,40]. A possible explanation is that the time point selected in this study (two weeks) may not coincide with peak TGF-β release, or that GCF levels do not fully reflect tissue-level cytokine dynamics [41,42]. Moreover, TGF-β is a multifunctional cytokine with roles in both inflammation and fibrosis, complicating its interpretation as a biomarker of regeneration.

Correlation analysis identified associations between higher EGF, FGF, and OPG levels and greater clinical improvements at 6 months. While these correlations are noteworthy, they should be interpreted cautiously given the complex and multifactorial nature of periodontal wound healing and the exploratory nature of the analysis.

Previous transcriptomic studies have identified gene modules associated with angiogenesis, osteogenesis, and odontoblast differentiation following EMD application, suggesting that EMD may facilitate early regenerative processes by upregulating endothelium-related genes involved in vascularization and mineralized tissue formation [43]. While our current analysis did not assess gene expression directly, the observed upregulation of growth factors such as FGF and EGF in GCF could reflect, at least in part, the activation of these pathways at the tissue level. However, the relationship between protein release in GCF and intracellular gene regulation remains speculative and requires further investigation. Also, proteomics techniques may help in elucidating more broadly the molecular events occurring at the site level [44,45,46].

Overall, this study demonstrates that adjunctive application of EMD in the flapless approach induces measurable changes in the local molecular environment within two weeks, specifically enhancing the expression of selected growth factors and bone metabolism regulators. These molecular findings support the potential role of EMD in promoting early periodontal healing processes.

However, it must also be considered that the gingival crevicular environment contains various proteolytic enzymes and zymogens capable of degrading enamel matrix proteins, potentially reducing the biological activity of EMD [47]. This underlines the importance of controlling local inflammation prior to EMD application, as a high protease burden may compromise the stability and effectiveness of the applied proteins. This concept aligns with findings in periodontal regenerative surgery, where the preoperative inflammatory status—particularly the reduction in bleeding on probing and inflammatory markers—has been shown to significantly influence clinical outcomes [48,49]. Accordingly, optimal inflammatory control before flapless intervention may be a prerequisite for maximizing the regenerative potential of EMD.

This study has several limitations. First, the relatively small sample size may limit the generalizability of the findings and the statistical power to detect subtle differences between the groups. Second, the short observational window does not allow for conclusions regarding the long-term stability of clinical and molecular outcomes. Finally, the sole reliance on GCF as a sampling medium may not capture all relevant cellular and molecular events occurring at the tissue level during periodontal regeneration. Further investigations using serial sampling and broader biomarker panels are needed to elucidate the temporal dynamics of EMD-induced molecular changes. In addition, future studies incorporating proteomic or transcriptomic approaches may provide deeper insights into the underlying mechanisms of periodontal wound healing.

## 4. Materials and Methods

### 4.1. Study Design and Population

The present study was designed as a parallel-group, double-blind, randomized controlled trial of 6-month duration and was performed according to the current standards of clinical research (CONSORT guidelines). The study protocol was approved by the institutional Ethics Committee of the A.O.U. Città della Salute e della Scienza di Torino. All participants signed an informed consent statement in accordance with the Declaration of Helsinki (1975, revised in 2013) before the start of the study. The trial was conducted at the Section of Periodontology, C.I.R. Dental School, University of Turin between June 2023 and March 2024.

Following completion of Steps I and II of periodontal therapy, patients with residual periodontal pockets were screened for inclusion. Eligibility criteria included: (1) diagnosis of stage III or IV periodontitis according to the 2017 Classification of Periodontal and Peri-Implant Diseases and Conditions; (2) full-mouth plaque and bleeding scores < 15%; (3) at least one residual pocket with PPD ≥ 6 mm and a radiographic intrabony component ≥ 3 mm; and (4) absence of systemic conditions or local factors contraindicating periodontal surgery. Smokers, pregnant and breastfeeding females, and patients with systemic diseases or under pharmacological therapy affecting periodontal healing were excluded. Teeth with a periapical lesion, furcation involvement, and mobility ≥ degree 1 were also excluded.

### 4.2. Randomization and Treatment Allocation

Patients were randomized using a balanced random permuted block approach. Allocation concealment was achieved using sequentially numbered, opaque, sealed envelopes prepared by an independent investigator not involved in the clinical procedures or data analysis. The sealed envelopes were placed into the custody of the study coordinator, who opened them just prior to the EMD application and informed the operator. Participants and outcome assessor were blinded to treatment assignment.

Experimental sites, one per each patient, were randomly allocated to:

– Test group: flapless approach with adjunctive application of EMD (Emdogain^®^, Straumann, Basel, Switzerland).
– Control group: flapless approach alone with simulated EMD application.

### 4.3. Flapless Procedure and Post-Operative Care

All procedures were performed by a single experienced clinician under magnification loupes (3.5–12.5×). Atraumatic lateral papilla displacement was used to access the defect, followed by debridement with ultrasonic tips and mini-curettes. Granulation tissue was carefully removed, and the root surface was thoroughly instrumented.

In the test group, the root was conditioned with 24% EDTA (PrefGel^®^, Straumann, Basel, Switzerland) for 2 min, rinsed with saline, and dried with sterile gauze. EMD was applied to the root surface using the manufacturer’s syringe. In the control group, all application steps were simulated, without releasing any gel. Gentle compression was applied to close the pocket, and no sutures were placed.

Participants were advised to avoid using the interproximal brush or flossing for only the first day. They were scheduled to visit every 2 weeks for the first month and every two months thereafter during the 6-month postoperative period. Individualized oral hygiene instructions were repeated at recall visits according to the patient’s needs, and no additional sub-gingival instrumentation was performed at the experimental sites.

### 4.4. GCF Sampling

GCF was sampled from the experimental site at baseline and 2 weeks post-treatment, prior to clinical measurements. After isolation with cotton rolls and supragingival plaque removal with a curette, the area was gently air-dried. Filter strips (PerioPaper^®^, Oraflow Inc., Sarasota, FL, USA) were gently inserted 1–2 mm subgingivally, allowing the strips to remain in place for 30 s. Blood-contaminated samples were discarded. The strips were placed into Eppendorf tubes containing 100 μL phosphate-buffered saline (PBS 1X pH = 7.6) and immediately stored at –80 °C until to be analyzed.

### 4.5. Determination of GCF Wound-Healing Mediator Levels

Total concentrations of selected biomarkers were determined using a high-sensitivity multiplex bead-based immunoassay (Bio-Plex^®^ Suspension Array System, Bio-Rad Laboratories, Hercules, CA, USA). The following analytes were quantified in each individual GCF sample:

– Epithelial repair: EGF.
– Connective tissue remodeling: MMP-8, FGF, TGF-β 1-2-3.
– Bone metabolism: OPG.

All assays were performed according to the manufacturer’s protocol. The GCF specimens and standards were eluted in assay buffer in 96-well filter plates. Micro-sphere beads coated with monoclonal antibodies against the markers were added. The plates were washed, and a mixture of biotinylated secondary antibodies was added. After a second round of incubation of 30 min the plates were washed again, and streptavidin conjugated to the fluorescent protein phycoerythrin was added. The plates were washed once more after 10 min to remove the unbound reagents, and Bio-Plex assay buffer was added to each well. Plates were analyzed using the Bio-Plex Manager Software version 6.2 (Bio-Rad Laboratories, Hercules, CA, USA), and results were reported as the total biomarker amount recovered from a 30 s GCF strip collection.

### 4.6. Clinical Parameters

At baseline and 6 months after treatment, a calibrated examiner, masked with respect to the treatment assignment, collected the clinical measurements at the deepest point of the selected defects by using a calibrated North Carolina manual periodontal probe (PCPUNC 15, Hu-Friedy, Chicago, IL, USA). The following clinical parameters were recorded: presence/absence of bacterial plaque, presence/absence of BoP, PPD, CAL and REC.

To perform the intra-examiner calibration, duplicate measurement of PPD and CAL were conducted on 12 non-study patients presenting with intrabony defects 48 h interval between each measurement. Intra-examiner weighted kappa values (±1 mm) were 0.91 for PPD and 0.88 for CAL.

### 4.7. Statistical Analysis

The primary outcomes were early changes in biochemical marker levels in GCF samples. The secondary outcomes were changes in clinical periodontal parameters throughout the 6-month study period.

Statistical analysis was performed using SPSS v28 (IBM Corp., Armonk, NY, USA). Normality was assessed with the Shapiro–Wilk test. Between-group differences in clinical and biomolecular variables were tested using the independent *t*-test or Mann–Whitney U test, as indicated. Adjustments for multiple comparisons were applied to reduce the risk of false-positive findings. Differences in categorical variables were assessed using the chi-square test or the Fisher exact test. Intragroup comparisons were performed with paired *t*-test or Wilcoxon signed-rank test. Correlations between biomarker levels and clinical parameters were explored using Spearman’s rank correlation. A *p*-value < 0.05 was considered statistically significant.

## 5. Conclusions

In conclusion, these preliminary findings suggest that the application of EMD in conjunction with the flapless approach may contribute to the early modulation of growth factors and bone metabolism-related markers in GCF. The observed increases in EGF and FGF are consistent with enhanced epithelial proliferation, fibroblast activity, and angiogenesis, while the elevated OPG levels suggest a protective effect against bone resorption. In contrast, EMD effect on MMP-8 suppression does not appear to exceed that achieved by debridement alone. These data provide insight into the molecular environment associated with early wound healing after EMD application, although their clinical relevance remains to be fully elucidated.

## Figures and Tables

**Table 1:** Baseline characteristics at patient and experimental site level.

Variables	Flapless (*n* = 19)	Flapless + EMD (*n* = 19)
Patient level		
Age (years; mean ± SD)	55.6 ± 7.5	53.1 ± 11.6
Males/females (*n*)	12/7	8/11
Light smokers/no smokers (*n*)	4/15	2/17
Periodontitis Stage III/IV (*n*)	9/10	7/12
FMPS (%; mean ± SD)	11.2 ± 2.6	11.9 ± 1.8
FMBS (%; mean ± SD)	12.5 ± 2.1	13.1 ± 1.6
Site level		
Gingival phenotype (thick/thin) (*n*)	18/1	16/3
Single-rooted/multi-rooted teeth (*n*)	11/8	9/9
Arch (maxilla/mandible) (*n*)	8/11	7/12
Mesial/Distal (*n*)	9/10	13/6

EMD, enamel matrix derivative; SD, standard deviation; FMPS, Full-mouth plaque score; FMBS, Full-mouth bleeding score.

**Table 2:** Baseline and 6-month clinical periodontal measurements at the experimental sites.

Variables	Baseline	6 Months	Δ Baseline-6 Months	*p*-Value Intragroup
REC (mm; mean ± SD)				
Flapless	0.6 ± 0.8	0.9 ± 1.0	0.3 ± 0.6	>0.05
Flapless + EMD	0.7 ± 0.9	0.8 ± 0.9	0.1 ± 0.4	>0.05
*p*-value intergroup		>0.05	>0.05	
PPD (mm; mean ± SD)				
Flapless	7.4 ± 1.3	4.8 ± 1.3	2.6 ± 0.8	**<0.001**
Flapless + EMD	7.2 ± 1.0	4.0 ± 1.2	3.2 ± 0.6	**<0.001**
*p*-value intergroup		**0.038**	**>0.05**	
CAL (mm; mean ± SD)				
Flapless	8.1 ± 1.2	5.8 ± 1.4	2.2 ± 1.2	**<0.001**
Flapless + EMD	7.9 ± 0.9	4.8 ± 1.5	3.1 ± 1.0	**<0.001**
*p*-value intergroup		**0.046**	**0.018**	

EMD, enamel matrix derivatives; SD, standard deviation; REC, gingival recession; PPD, probing pocket depth; CAL, clinical attachment level. Statistically significant differences are shown in bold.

## Data Availability

All data supporting the findings of this study, not restricted by privacy regulations, are reported within the manuscript.

## Abbreviations

The following abbreviations are used in this manuscript:

Abbreviation	Full Term
BoP	Bleeding on Probing
CAL	Clinical Attachment Level
EGF	Epidermal Growth Factor
EMD	Enamel Matrix Derivative
FGF	Fibroblast Growth Factor
FMBS	Full-Mouth Bleeding Score
FMPS	Full-Mouth Plaque Score
GCF	Gingival Crevicular Fluid
MIST	Minimally Invasive Surgical Technique
MMP-8	Matrix Metalloproteinase-8
MINST	Minimally Invasive Non-Surgical Treatment
OPG	Osteoprotegerin
PBS	Phosphate-Buffered Saline
PI	Plaque Index
PPD	Probing Pocket Depth
REC	Gingival Recession
RANKL	Receptor Activator of Nuclear Factor Kappa-B Ligand
TGF-β	Transforming Growth Factor-beta
